# Neuronal maturation reduces the type I IFN response to orthobunyavirus infection and leads to increased apoptosis of human neurons

**DOI:** 10.1186/s12974-019-1614-1

**Published:** 2019-11-18

**Authors:** Clayton W. Winkler, Tyson A. Woods, Bradley R. Groveman, Aaron B. Carmody, Emily E. Speranza, Craig A. Martens, Sonja M. Best, Cathryn L. Haigh, Karin E. Peterson

**Affiliations:** 1 Laboratory of Persistent Viral Diseases, Rocky Mountain Laboratories, National Institute of Allergy and Infectious Diseases, National Institutes of Health, 903 S. 4th Street, Hamilton, MT 59840 USA; 2 Research Technologies Branch, Rocky Mountain Laboratories, National Institute of Allergy and Infectious Diseases, National Institutes of Health, Hamilton, MT 59840 USA; 3 Laboratory of Virology, Rocky Mountain Laboratories, National Institute of Allergy and Infectious Diseases, National Institutes of Health, Hamilton, MT 59840 USA

**Keywords:** Orthobunyavirus, Encephalitis, Human cerebral organoids, Neurogenesis, Neuron, Type I interferon, Apoptosis, Single-cell transcriptomics

## Abstract

**Background:**

La Crosse virus (LACV) is the leading cause of pediatric arboviral encephalitis in the USA. LACV encephalitis can result in learning and memory deficits, which may be due to infection and apoptosis of neurons in the brain. Despite neurons being the primary cell infected in the brain by LACV, little is known about neuronal responses to infection.

**Methods:**

Human cerebral organoids (COs), which contain a spectrum of developing neurons, were used to examine neuronal responses to LACV. Plaque assay and quantitative reverse transcription (qRT) PCR were used to determine the susceptibility of COs to LACV infection. Immunohistochemistry, flow cytometry, and single-cell transcriptomics were used to determine specific neuronal subpopulation responses to the virus.

**Results:**

Overall, LACV readily infected COs causing reduced cell viability and increased apoptosis. However, it was determined that neurons at different stages of development had distinct responses to LACV. Both neural progenitors and committed neurons were infected with LACV, however, committed neurons underwent apoptosis at a higher rate. Transcriptomic analysis showed that committed neurons expressed fewer interferon (IFN)-stimulated genes (ISGs) and genes involved IFN signaling in response to infection compared to neural progenitors. Furthermore, induction of interferon signaling in LACV-infected COs by application of recombinant IFN enhanced cell viability.

**Conclusions:**

These findings indicate that neuronal maturation increases the susceptibility of neurons to LACV-induced apoptosis. This susceptibility is likely due, at least in part, to mature neurons being less responsive to virus-induced IFN as evidenced by their poor ISG response to LACV. Furthermore, exogenous administration of recombinant IFN to LACV COs rescued cellular viability suggesting that increased IFN signaling is overall protective in this complex neural tissue. Together these findings indicate that induction of IFN signaling in developing neurons is an important deciding factor in virus-induced cell death.

## Background

La Crosse virus (LACV) is an arthropod-borne orthobunyavirus endemic to North America [1]. It was first isolated from the brain of a patient that died of pediatric encephalitis in 1960 [2] and is currently the leading cause of pediatric encephalitis in the USA [3]. Symptoms include headache, fever, and vomiting progressing to lethargy, behavioral changes, and/or brief seizures until resolution 7–8 days after onset [4–6]. In severe cases, neurological symptoms present more quickly and can progress to deep coma. Of symptomatic cases, ~ 50% develop seizures with status epilepticus occurring in 10–15%. The case fatality rate is low at ~ 1%, however, long-term sequelae can occur. One study demonstrated abnormal behaviors and EEG readings in 15% of patients 1–5 years following hospital discharge [7]. Another study reported 32% of patients with severe LACV encephalitis performed lower than expected on intelligence testing administered 12–18 months post-hospitalization [5] suggesting long-term learning impairment. Despite the clinical importance of this virus, little is known about its pathogenesis in humans.

Several histopathological studies from post-mortem and biopsied human tissues have defined hallmarks of LACV encephalitis that include infection, damage, and degeneration of neurons [2, 4]. However, these studies failed to define whether specific developing neuronal populations were infected or whether infection and/or death of neuronal cells is maturation-dependent. Originally, cultured post-mitotic neurons were found to be more resistant to LACV-induced apoptosis than undifferentiated progenitor cells [8] leading to the dogma that mature adult neurons are resistant to LACV-induced cell death. However, a recent study using human neuronal/astrocyte co-cultures suggests that differentiated neurons could be infected with LACV and undergo cell death [9]. To directly examine if neuronal maturity affects the ability of LACV to infect and/or induce apoptosis of neurons requires a system with neurons at different stages of maturity. This is particularly important for LACV infection, as LACV primarily causes disease in young children, with the main age range of 4 to 11, a time point where the brain is still undergoing active neurogenesis [10]. Furthermore, one of the clinical signs associated with LACV infection is learning and memory impairment, processes that are thought to involve hippocampal neurogenesis [11, 12]. Thus, understanding how LACV may impact neurons at different stages of maturity is critical for understanding disease pathogenesis.

Induced pluripotent stem cell (iPSC)-derived human cerebral organoids (COs) are a highly useful tool in examining virus infection of neurons in that they contain a spectrum of developing neurons within the same complex, interconnected tissue. They exhibit a similar inside-out patterning and regional organization as a developing human brain [13–15], with ventricular zone-like regions populated by SOX2 expressing neural progenitors that give rise to Doublecortin (DCX) expressing immature neurons. These immature neurons are committed to the neuronal lineage [16] and migrate radially away from the neurogenic regions to populate and integrate into the tissue as Class III β-tubulin (βIII-tubulin) expressing mature neurons [17, 18]. Thus, COs are a unique model in which to study LACV pathogenesis as they contain neurons at varying stages of development and connectivity. In this study, we utilize COs to model viral tropism in neuronal cells at various developmental stages and determine if the developmental stage of neurons influences virus-induced cell death. Additionally, we analyze differences in gene expression between neuronal populations to determine if neuronal maturity affects the response to viral infection.

## Methods

### iPSC-derived human cerebral organoid generation and maintenance

Cerebral organoids (COs) were generated using the protocol and media formulations described in Lancaster and Knoblich [17], with the following minor variations. Source tissue was KYOU-DXR0109B human-induced pluripotent stem cells (iPSC; ATCC). Embryoid bodies (EBs) were generated using one 25 cm^2^ flask of cells to seed a 96-well low-adhesion U-well plate. EBs were transferred into neural induction medium between days 4 and 6 depending upon morphology. Organoids were Matrigel embedded on day 3 or 4 post neural induction. Four days after embedding, organoids were transferred into an agitated culture in upright 25 cm^2^ low-adhesion flasks on an orbital shaker (Heathrow Scientific) at 85 rpm. Media was changed 2–3 times per week until 21 days post neural induction when COs were plated individually into low-adhesion 24 well plates for infection. The agitated culture was maintained, and media completely changed/collected daily.

### Virus stocks and infection

LACV stocks were generated as previously described [19]. The virus for CO infection was diluted to either 10^3^ or 10^1^ plaque-forming units (PFU) in 500 μl of medium. Medium from wells containing COs was removed and replaced with a virus-containing medium for 2 h in the incubator while shaking. After infection, wells containing COs were washed and refilled with 1 ml fresh medium.

### Treatment of COs with recombinant IFN

COs were infected and maintain in culture as described above except that beginning at 1 dpi (day post-infection), recombinant human IFNβ1 or IFNα2 and IFNα4 (PBL Assay Science) or vehicle (1×PBS with 0.1% fetal bovine serum) was added to each daily media change. All recombinant IFNs were used at a dose of 1000 U/ml. An equivalent volume of vehicle solution was used for control samples.

### RNA isolation and quantitative reverse transcription (qRT) PCR from COs and media supernatant

At indicated time points, either whole or half COs or cell culture medium was isolated, flash-frozen, and stored at −80°C. COs were dissociated by incubation in 0.5 ml of Corning™ Cell Recovery Solution for 1 h at 4°C. Samples were washed 3 times with 1×PBS by pelleting at 500 g and then placed into ZR RNA buffer for RNA isolation and clean up according to the manufacturer’s protocols (Zymo Research). Viral RNA was isolated from CO culture supernatants using the *Quick*-RNAViral RNA Kit (Zymo Research) according to the manufacturer’s protocols. Analysis of mRNA from COs was performed as previously described [20]*.* Viral RNA in culture media was determined by fitting sample *C*_*T*_ values to a standard curve obtained from a linear regression fit from 10-fold dilutions of spiked cell culture media containing known virus PFUs. Primers used: hGapdh.2-707F 5′-TCGTGGAAGGACTCATGACC-3′, hGapdh.2-818R 5′-ATGATGTTCTGGAGAGCCCC-3′ and LACV.2-552F 5′-ATTCTACCCGCTGACCATTG-3′, LACV.2-650R5’-GTGAGAGTGCCATAGCGTTG-3′.

### Replication kinetics

COs were infected with LACV as described above and culture supernatants were harvested at 1–6 dpi. Supernatants were titered as described previously [21]*.*

### Cell viability assay

Indicated COs were assayed daily for cell viability using PrestoBlue™ (Molecular Probes). Daily, culture medium was removed from each CO-containing well and replaced with 0.5 ml of 1×PrestoBlue™ reagent in medium for a 30 min incubation. Samples and controls were read in triplicate using fluorescence excitation at 560 nm and emission at 590 nm. Data are reported as percent 590 nm fluorescence relative to a 0 dpi baseline taken for each CO.

### Immunohistochemistry labeling and quantification

Prior to tissue processing, some COs were treated according to the manufacturer’s protocol with a fixable SR-VAD-FMK poly caspase probe (ImmnoChemistry Technologies) to detect activated caspases. At indicated time points, either whole or half COs were collected from culture and placed in 10% neutral buffered formalin for 1–3 days and protected from light. COs were prepared, cryosectioned, and immunolabeled as previously described [22]. Primary antibodies used were LACV (in-house generated polyclonal mouse, 1:500) and active caspase 3 (Promega#G7481, 1:250) or Sox2 (abcam #97959, 1:1,000 or DCX (abcam #18723, 1:500) or βIII tubulin (abcam #18207, 1:5000) or NeuN (abcam #104225, 1:2000). Secondary conjugated antibodies used goat anti-mouse 488, donkey anti-rabbit 594 or donkey anti-rabbit 647 (Molecular Probes, 1:500). High-resolution images were taken on a Zeiss Axio Scan.Z1 with the × 40 objective or on a Zeiss 710 LSM with the × 63 objective. For quantification, thresholds for Sox2, DCX, or βIII tubulin positive signal labeling in whole-organoid scans were determined, and the area of positive signal was obtained. This area was then converting into a percent area by dividing by the area of the entire section as determined by staining of cell nuclei and an overlaid brightfield image. Thresholds for positive signal was determined to be 1.25× the average pixel intensity obtained from secondary only labeled control tissues and above. Images were processed in Imaris v8.4.1 or Fiji.

### Single-cell RNA sequencing and analysis

Mock and infected COs were dissociated as described above with the exception that washes and collections were done in 1× PBS with 1% bovine serum albumin. Cells were counted and diluted to 10^6^/mL prior to loading onto the 10× Genomics Chromium Controller instrument to be individually partitioned into nanoliter scale droplets within an oil emulsion. RNA sequencing libraries were constructed using the Chromium Single cell 3′ Reagent kit V2 (10× Genomics PN-120237) and assayed using a Bioanalyzer 2100 (Agilent) according to the respective manufacturer’s instructions. Samples were sequenced using a HiSeq 2500 with a Rapid Paired-End flow cell and cluster kit and Rapid SBS chemistry for a 26/8/98 cycle run (Illumina). Sequencing reads were demultiplexed and mapped to Human Genome build GRCh38.p5 using the Cell Ranger software package (version 2.1.0) from 10× Genomics. Downstream single-cell RNA-Seq analysis was performed using the R software package Seurat (version 2.3.1) [23]. Briefly, canonical correlation analysis (CCA) was performed on data from the two sample types: mock and LACV COs. CCA subspaces were combined for t-distributed stochastic neighbor embedding (t-SNE) analysis, clustering, and visualization.

### Flow cytometry

Mock and infected COs were dissociated as described above and labeled with Fixable Viability Dye eFluor780 (eBioscience, 1:1000) for 30 min in the dark on ice. Intracellular and nuclear labeling and data collection and analysis were performed as previously described [24]. Antibodies used: Trustain FcX™ (Biolegend #422301, 1:1000), Sox2 (Millipore #FCMAB112, 1:50), DCX (BD Pharmigen #561505, 1:50), and βIII tubulin (R&D Systems #IC1195C, 1:50).

### Statistical analysis

All statistical analyses were carried out in Prism 7.0c and are described in the figure legends.

## Results

### iPSC-derived human cerebral organoids are permissive to LACV infection and replication

**Fig. 1:**
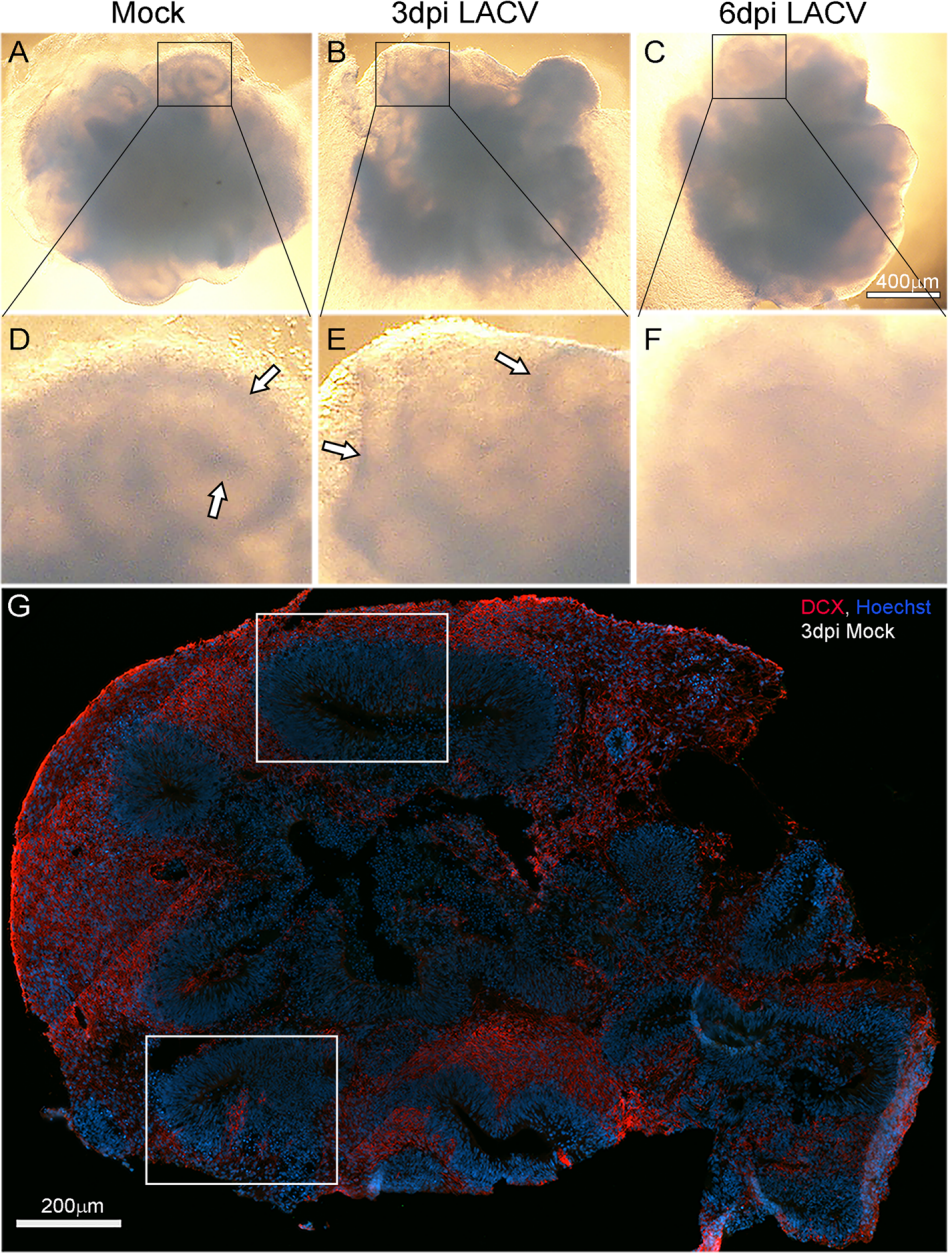
Morphology of cerebral organoids during LACV infection. Representative brightfield images of **a** mock, **b** 3 dpi LACV, and **c** 6 dpi LACV COs. **d-f** Enlargements of boxed areas in **a-c** are shown. White arrows in **d** and **e** indicate areas of structural complexity that appear diminished by 6 dpi (**f**). Scale bar in **c** corresponds to **a-f**. **g** Representative immunofluorescence scanned image of a whole-mount, mock-infected cerebral organoid labeled with doublecortin (DCX, red) and Hoechst (blue) to label committed neurons and nuclei respectively. Areas in white boxes are analogous to those highlighted in **a–f** and are similar to areas focused on in subsequent experiments

The consequence and outcomes of LACV infection in human neural tissue are poorly studied. Thus, we infected human-derived iPSC cerebral organoids (COs) with 10^3^ or 10^1^ PFU of the virus isolated from post-mortem human brain tissue [25]. Visually, the overall size of infected COs did not change over time (Fig. 1a–c). However, structural density and complexity appeared to be reduced by 6 dpi in the 10^3^ PFU (Fig. 1c, f), but not in the 10^1^ PFU (not shown) or mock-infected COs (Fig. 1a, d, white arrows). Structural features were still observed at 3 dpi in COs infected with 10^3^ LACV (Fig. 1b, e, white arrows). In histological samples, these areas of structural complexity correlated with nuclei-dense ventricular zone-like regions surrounded by cells undergoing neurogenesis as indicated by Doublecortin (DCX) expression (Fig. 1g). These regions (Fig. 1g, white boxes) became the focus for subsequent histological analysis.

**Fig. 2:**
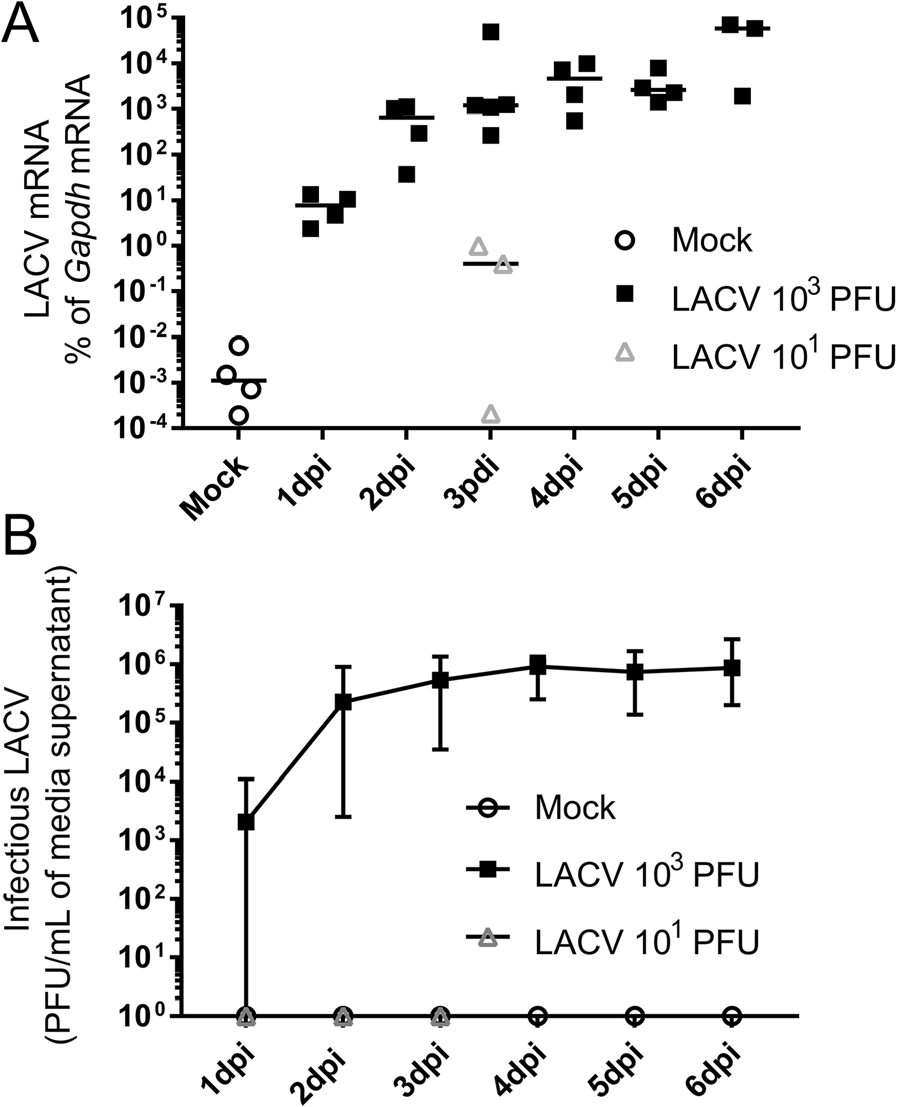
Replication kinetics in LACV-infected iPSC-derived human COs. **a** COs were collected at indicated timepoints for evaluation of viral RNA via qRT PCR with virus-specific primers. Viral RNA levels for each sample were calculated as the percentage difference in threshold cycle (*C*_*T*_) value (Δ*C*_*T*_ = *C*_*T*_ for *Gapdh* gene-*C*_*T*_ for virus). Viral mRNA was plotted as the percentage of gene expression relative to that of the *Gapdh* gene. Open circles indicate mock COs, closed squares COs infected with 10^3^ PFU, and open squares CO infected with 10^1^ PFU. Each point represents one CO. **b** Media supernatants were collected from individual wells containing COs at the indicated time point and assayed for PFU/mL by plaque assay. Data are representative of *n* = 6–12 mock, *n* = 6–24 LACV 10^3^, and *n* = 3 LACV 10^1^ COs

To determine viral replication kinetics in COs, quantitative real-time (qRT) PCR analysis and plaque assays were performed. LACV RNA expression in COs infected with the 10^1^ PFU LACV showed weak positive signal in 2 of 3 COs (Fig. 2a), but no detectable infectious virus in media supernatants by plaque assay (Fig. 2b) demonstrating little-to-no replicating virus. In contrast, COs infected with a 10^3^ PFU dose showed an ~ 10-fold daily increase in LACV RNA expression from 1 to 3 dpi compared to *Gapdh* reference (Fig. 2a). Relative viral RNA expression remained stable in infected COs from 3 to 6 dpi suggesting viral replication had peaked by 3 dpi. Similarly, viral titers in media supernatants from COs infected with 10^3^ PFU LACV peaked and plateaued at 3 dpi, remaining stable through 6 dpi (Fig. 2b). These findings demonstrate human iPSC-derived COs are susceptible to infection by LACV and support viral replication at a 10^3^ PFU dose, causing reduced organoid structural complexity by 6 dpi. Thus, all subsequent infections were performed with 10^3^ PFU of LACV.

### Cells in LACV-infected iPSC-derived human COs undergo caspase3-dependent cell death

**Fig. 3:**
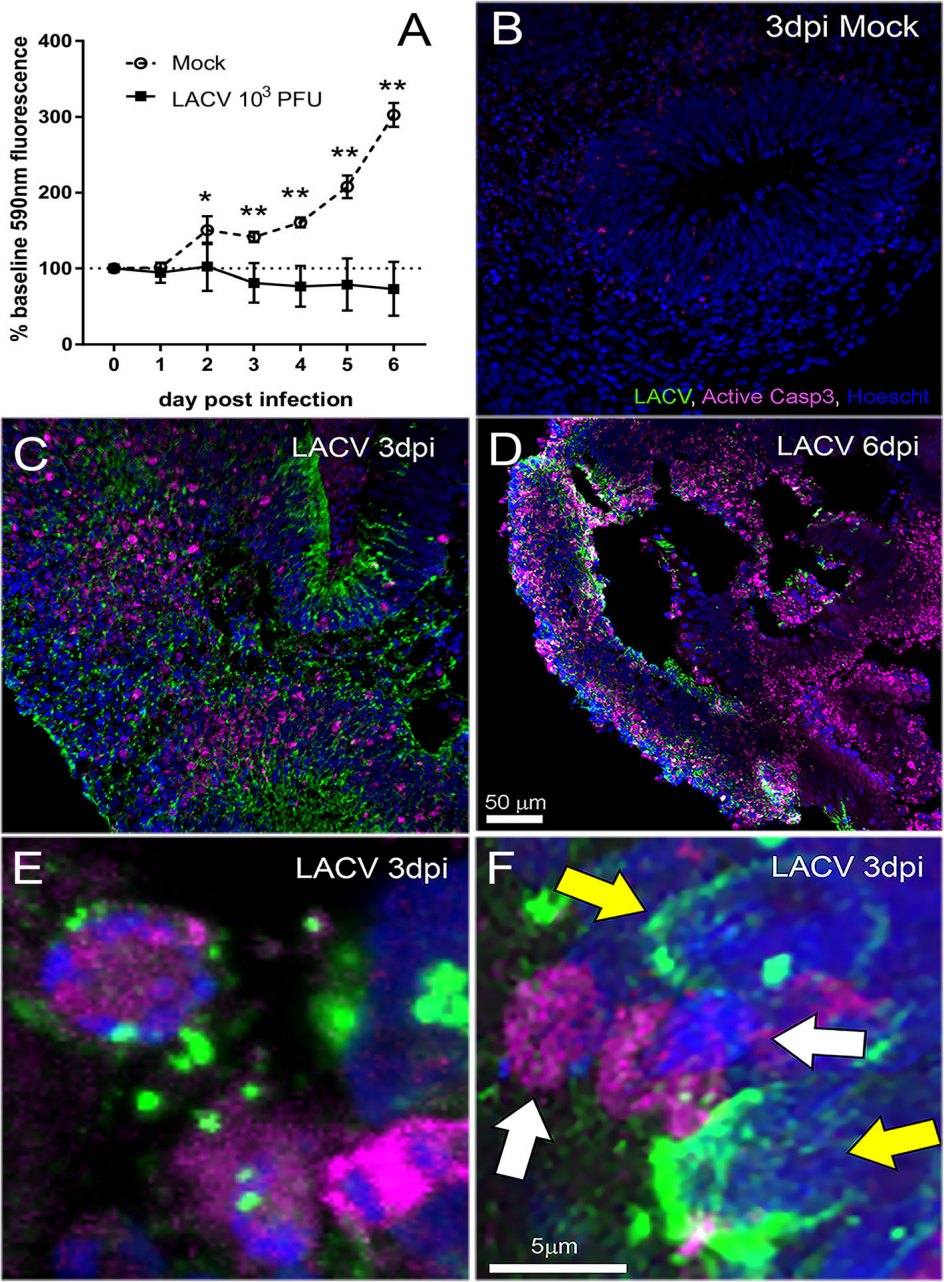
Cell death in LACV-infected iPSC-derived human COs. **a** A resazurin reduction-based cell viability assay was performed daily on individual COs within a 24 well plate. Data is plotted as an average percent of base fluorescence measured at 590 nm for each CO just prior to infection with mock (open circles) or LACV (closed squares). Fluorescence for each sample at each time point was read in triplicate at the indicated time point. A two-way ANOVA with a Sidak’s multiple comparisons test was used to determine significance. *p < 0.05, **p < 0.001. Data are representative of *n* = 3 mock and *n* = 6 LACV 10^3^ COs. **b–d** Representative images of COs immunohistochemically labeled for active caspase-3 (Casp3, magenta), LACV (green), and nuclei (blue). **b** is from a mock-infected CO, **c** a 3 dpi LACV-infected organoid and **d** a 6 dpi LACV-infected organoid. Scale bar in **d** also applies to **b** and **c**. **e**, **f** High magnification images taken from the same section of a 3 dpi LACV-infected CO. **e** Illustrates a caspase3^+^ cell (magenta) that is infected with LACV (green). **f** Caspase3^+^ cells that are not infected with LACV (white arrows) and LACV-infected cells that are not caspase3^+^ (yellow arrows). Scale bar in **f** also applies to **e**

To examine whether LACV infection of the organoid resulted in neuronal death, we monitored cellular viability of mock- and LACV-infected COs through time using a resazurin reduction-based colorimetric assay. Mock-infected samples converted increasing amounts of resazurin substrate compared to baseline readings throughout experiments (Fig. 3a), suggesting these COs were still growing, although they did not visually appear to increase significantly in size. In contrast, LACV-infected COs converted equivalent or decreasing amounts of resazurin substrate during the experiments compared to baseline (Fig. 3a), with significantly less conversion of resazurin than mock-infected COs by 3 dpi. The decreased amount of resazurin substrate conversion in LACV-infected COs indicated these COs had less cellular metabolism which could be a result of cell death.

LACV infection has been shown to induce neuronal cell death through an apoptotic mechanism in both mouse and human neural cell lines [8, 9, 26]. To directly examine apoptosis, we analyzed COs by immunohistochemistry for active caspase-3 and LACV antigen. Supporting the observed reduction of structural complexity (Fig. 1a–f) by LACV infection, Hoechst nuclear staining showed clear structural features in mock COs (Fig. 3b), but a reduction in these features in LACV-infected COs at 6 dpi (Fig. 3d). Active caspase-3 antigen could be found in a few cells in mock-infected COs at 3 dpi (Fig. 3b), indicating a low level of apoptosis in these organoids in the absence of infection. In contrast, widespread staining for active caspase-3 was found in LACV-infected organoids at 3 dpi (Fig. 3c) and was even more pronounced at 6 dpi (Fig. 3d). Active caspase-3 antigen was commonly found in LACV-infected cells (Fig. 3e) but was also found in cells that were negative for LACV suggesting infection can have a negative bystander effect on human neurons (Fig. 3f, white arrows). Conversely, some cells appear to be infected with LACV, but did not express active caspase-3 antigen suggesting some cells may be resistant to LACV-induced apoptosis (Fig. 3f, yellow arrow).

### LACV infection-induced apoptosis occurs more frequently in committed neurons

**Fig. 4:**
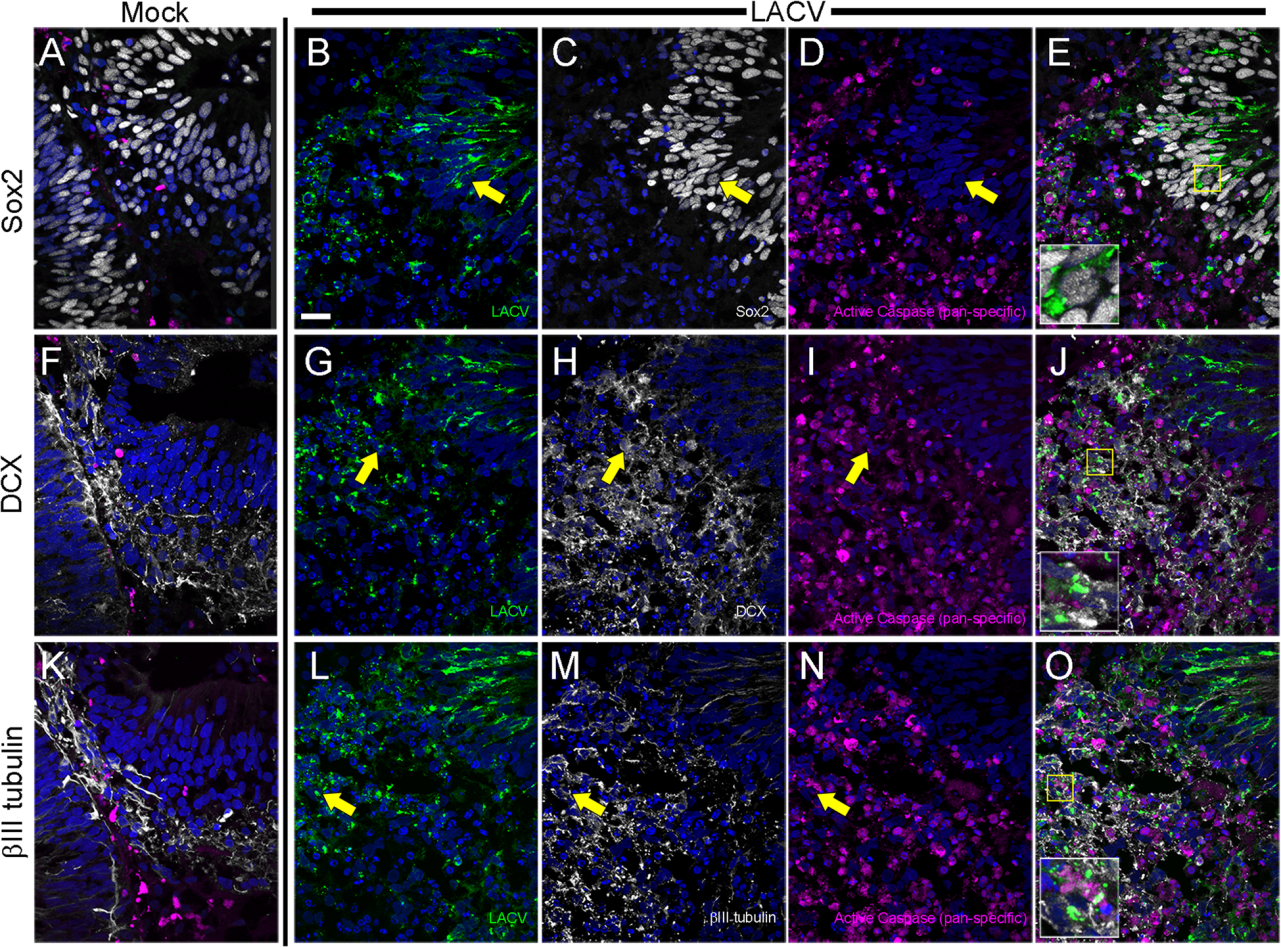
Committed neurons are susceptible to LACV-induced apoptosis. Representative confocal images of (**a**, **f**, and **k**) mock or (**b**–**e**, **g**–**j,** and **l**–**o**) 3 dpi LACV-infected COs immunohistochemically labeled with LACV (green), neuronal phenotyping antibodies (white), activated poly caspases (magenta), and nuclei (blue). Images are grouped by row using the neuronal phenotyping antibody with Sox2 being top, DCX middle, and βIII tubulin bottom. The three middle columns are images of single channels overlaid on nuclei from 3 dpi LACV-infected COs that are labeled accordingly. The far-right column (**e**, **j**, and **o**) is a combination of all four labels. The insets in **e**, **j**, and **o** are enlarged images of the highlighted yellow boxes in each panel. The corresponding yellow arrows in the associated individual label panels highlight the cell of interest shown in the inset. The images in the **a**, **f**, and **k** mock column are a combination of all four labels. All images were taken with a × 63 objective and are maximum intensity projects of 3 μm *z*-stacks taken with a 0.5 μm step. Scale bar in *B* = 20 μm and applies to all panels

To directly examine if there was a difference in which neural developmental stages were being infected and/or undergoing apoptosis, we labeled mock- and LACV-infected COs at 3 dpi with FLICA SR-VAD-FMK reagent to detect activated, pro-apoptotic caspases within cells, as well as individual antibodies used to detect virus and neural-lineage cells at distinct development stages (Fig. 4). Sox2 expression was used as a marker of neural progenitor cell, Doublecortin (DCX) for immature committed neurons, and class III β-tubulin (βIII-tubulin) for mature neurons. All three of these labels have been used previously to label respective neuronal populations in human iPSC-derived COs [13]. Mock-infected COs contained populations of all three groups of cells with relatively few cells staining for active pro-apoptotic caspases (Fig. 4a, f, k). In LACV COs, Sox2^+^ progenitor cells (Fig. 4a) were commonly infected (LACV, green), but were rarely positive for active caspases (magenta) at 3 dpi (Fig. 4b–e, inset representative of Sox2^+^ cell in infected CO). In the same organoids, DCX^+^ (Fig. 4g–j,) and βIII-tubulin^+^ (Fig. 4l–o) cells were commonly associated with areas of dense activated caspase labeling and LACV antigen. Individual DCX^+^ (Fig. 4j, inset) and BIII-tubulin^+^ (Fig. 4o, inset) cells were commonly found to be positive for both LACV and active caspases, suggesting these cells were actively undergoing apoptosis.

### Cells expressing committed neuron transcriptome profiles are reduced in LACV-infected COs

**Fig. 5:**
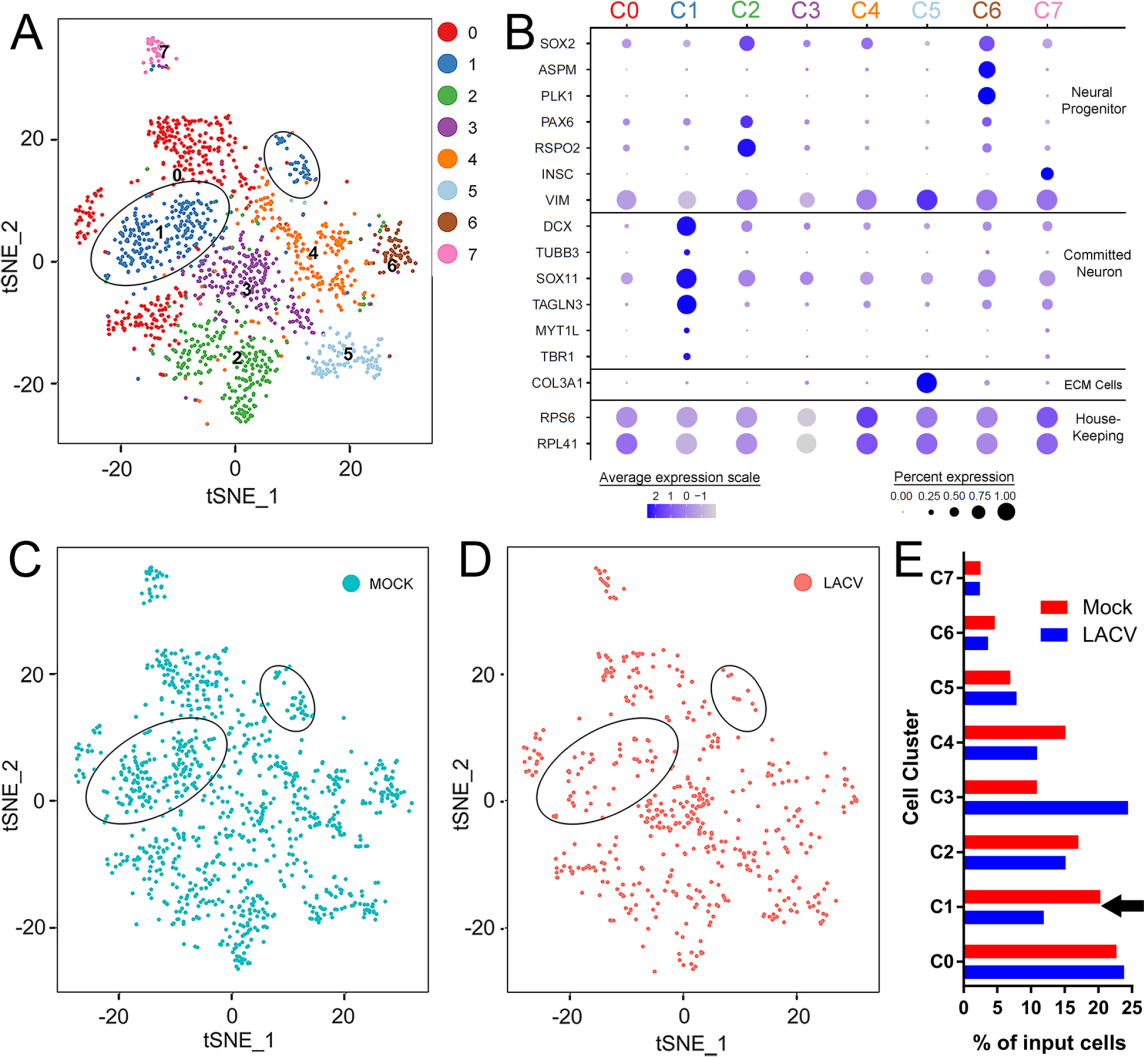
Single-cell transcriptomic profiling of mock- and LACV-infected organoids. **a** t-distributed stochastic neighbor embedding (tSNE) plots of canonical correlation analysis (CCA) aligned cell transcript profiles from 3 dpi mock- and LACV-infected COs clustered according to relatedness. Seven clusters were identified for optimal data resolution according to CCA and each is indicated by a different color. Each dot within each cluster represents the transcript profile from an individual cell. **b** Neural progenitors, committed neurons or extracellular matrix (ECM) producing mesenchymal cell-related transcript expression are shown for each identified cluster in **a**. Ribosomal (house-keeping) transcript expression in each cluster is shown as a positive control for sequencing. Increasing expression of each transcript is illustrated according to the deepening shade of gray to purple to blue. The proportion of cells expressing each transcript within the cluster is indicated by the size of the dot (scales at bottom). The tSNE plot in **a** was split to show cell transcript profiles from the (**c**) mock- or (**d**) LACV-infected CO still arranged within the 7 clusters. The two ellipses on each plot represent the boundaries of C1. The proportion of input cell transcript profiles in each cluster (**e**) is shown for both the mock (red) and LACV (blue) CO. The arrow in **e** denotes the decrease observed in C1 with LACV infection

Single-cell transcriptomic analysis has previously been used to identify neural-lineage cells of differing developmental stages in human cerebral organoids [27]. Therefore, single-cell RNA sequencing was performed on 3 dpi mock- and LACV-infected COs. Sequences from 1040 cells from mock and 496 cells from LACV COs were computationally aligned and integrated into 7 clusters (C1-C7) using canonical correlation analysis (CCA) and plotted using t-distributed stochastic neighbor embedding (tSNE) to visualize cellular transcript relatedness in 2D (Fig. 5a). Expression of neural progenitor and committed neuron transcripts was examined within each cluster of this aligned population to determine the predominate cellular development stage (Fig. 5b). Three clusters (C0, C3, and C4) had transcriptomic profiles that could not be easily classified and were not consistent with neural progenitor or committed neuron gene profiles. Another cluster, C5, expressed high levels of *COL3A1* consistent with extracellular matrix-producing mesenchymal cells that provide structure to COs [27]. Clusters C2, C6, and C7 primarily contained cells expressing transcripts such as *SOX2*, *ASPM*, *PLK1*, *PAX6*, *RSPO2*, *INSC*, and *VIM* which have been shown to be associated with neural progenitors [27–33]. Cluster C1 primarily expressed high levels of transcripts such as *DCX*, *TUBB3* (βIII tubulin), *SOX11*, *TAGLN3*, *MYT1L*, and *TBR1* and decreasing amounts of *VIM* which have been associated with cells undergoing neurogenesis and committed neurons [16, 27, 32–34]. Thus, single-cell analysis clearly identified and segregated neural lineage cells of differing developmental stages into these clusters with both neuronal progenitor (C2, C6, C7) and committed neuron (C1) populations.

To determine whether LACV infection-induced apoptosis (Figs. 3 and 4) resulted in cellular dropout in specific neural-lineage subpopulations, cells input from mock (Fig. 5c) or LACV (Fig. 5d) COs were identified within the 2D alignment and plotted on two separate tSNE plots, still grouped within their assigned clusters. Cells from individual clusters were then quantified as a percent of input cells from mock or infected COs (Fig. 5e). The percent of input cells in the neural progenitor clusters (C2, 6, and 7) were similar between mock and LACV samples demonstrating equivalent proportions of neural progenitors in both samples. In contrast, the percentage of cells in the committed neuron (C1) cluster was decreased by 41% in the infected sample compared to the mock (Fig. 5c, d, ellipses and e, black arrow). Thus, LACV infection of COs reduced the number of cells within C1 but not within the clusters corresponding to cells expressing neural progenitor transcripts.

### Quantitative analysis of LACV infection-induced caspase activity within neural-lineage subpopulations of human COs

**Fig. 6:**
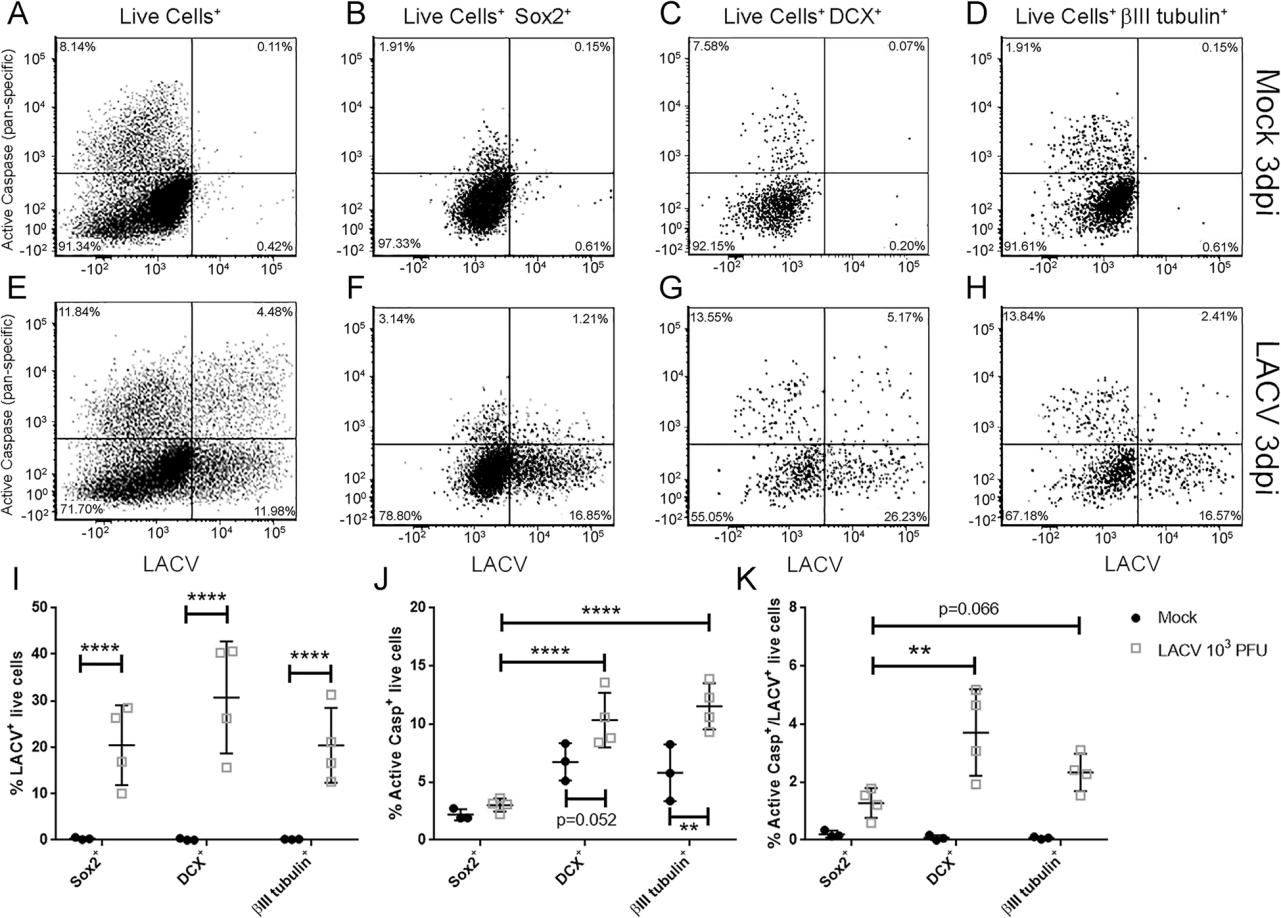
Flow cytometry analysis of neuronal cells from mock- and LACV-infected COs. Mock- (**a**–**d**) and LACV-infected (**e**–**h**) COs were non-enzymatically digested into a single cell suspension and analyzed via flow cytometry as described in the “Methods” section. Two representative examples are shown. Live cells were identified and interrogated for expression of activated poly-caspases (*y*-axis) and LACV expression (*x*-axis). **a**, **e** Active caspase and LACV staining from the whole live cell population. Gating within the whole live cell population for Sox2 (**b**, **f**), DCX (**c**, **g**) and βIII tubulin-positive cells allowed for examination of active poly-caspase and LACV staining within each neuronal population. Proportions of LACV-infected (**i**), activated poly-caspase (**j**), and LACV-infected/activated poly-caspase double-positive (**k**) neuronal cells within mock (closed circles) or infected (open squares) COs are shown. A two-way ANOVA with a Sidak’s multiple comparisons test was used to determine significance. **p < 0.001, ****p < 0.0001

To directly quantify the level of apoptosis in different neuronal populations following LACV infection, cells from mock and infected COs were isolated and analyzed using flow cytometry. COs labeled with FLICA SR-VAD-FMK reagent to detect apoptotic cells were non-enzymatically dissociated and labeled with a live-dead stain and antibodies to detect virus and neural-lineage phenotypes. Gating on live cells of specific neural-lineage phenotypes demonstrated that mock samples (Fig. 6a–d) contained some apoptotic cells (Fig. 6a, top left quadrant) as had been observed immunohistologically (Fig. 3b), but no LACV positive (Fig. 6a, lower right quadrant) or active caspase^+^/LACV^+^ double-positive cells (Fig. 6a, top right quadrant). In contrast, infected COs (Fig. 6e–h) had increased proportions of all three of these groups relative to mock (Fig. 6e). Gating on Sox2^+^ neural progenitor cells (Fig. 6b, f), DCX^+^ immature neurons (Fig. 6c, g), and βIII-tubulin^+^ mature neurons (Fig. 6d, h) in mock or infected COs allowed for comparison of caspase activation and LACV infection within these neural-lineage subpopulations. Plotting of individual COs showed similar proportions of LACV-infected cells between each subpopulation in infected COs (Fig. 6i). However, proportionally more committed neurons (DCX^+^ or βIII tubulin^+^) were positive for active caspase than neural progenitors (Sox^+^) in infected COs (Fig. 6j). Additionally, the proportions of active caspase positive committed neurons in infected COs were increased relative to mock controls demonstrating virus-specific apoptosis of these neuronal populations (Fig. 6j). Gating on LACV^+^ cells also showed more apoptosis in committed neurons compared to neural progenitors, particularly within the DCX^+^ population (Fig. 6k). Further complementing this finding, the overall number of DCX^+^ and βIII-tubulin^+^ committed neurons was reduced in LACV-infected COs compared to mock COs (Fig. 6g, h vs. c, d, bottom left quadrants). In contrast, the number of neural progenitors was relatively similar between mock and infected (Fig. 6f vs b, bottom left quadrant). Together, these data suggest that committed neurons are more susceptible to LACV-induced apoptosis than neural progenitors.

### The type I interferon response to LACV is more robust in neural progenitors than committed neurons

**Fig. 7:**
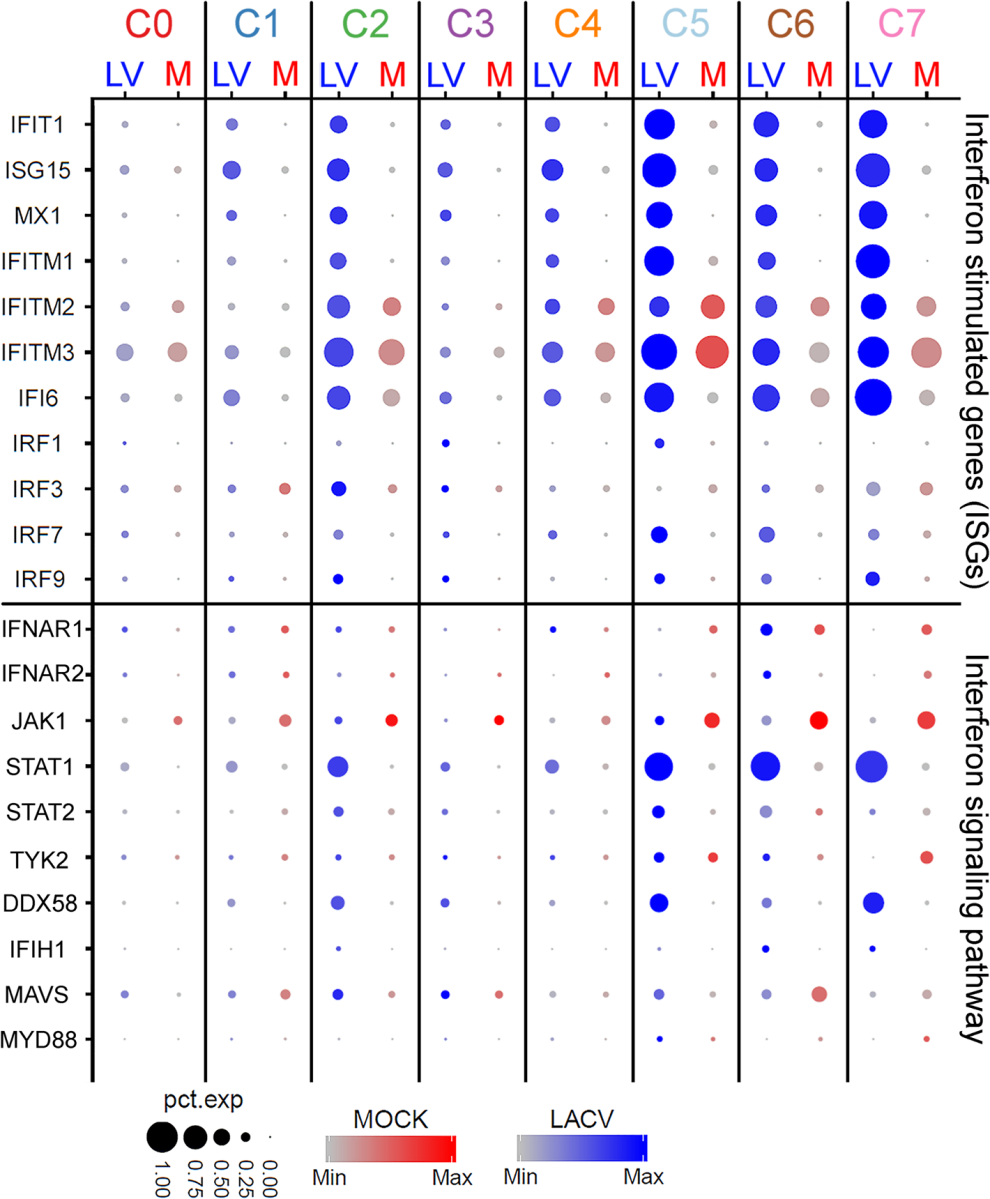
The type I interferon (IFN) response to LACV in neural progenitors and committed neurons. Expression of interferon-stimulated gene (ISG, upper section) type I IFN signaling and effector transcripts in the mock and LACV cells from COs that underwent single-cell transcriptome analysis. The same 7 clusters that were identified in Fig. 5 are shown and are segregated into the mock (red) and LACV (blue) samples. Increasing expression of each transcript is illustrated according to the deepening shade of either red for mock or blue for LACV. The proportion of cells expressing each transcript within the cluster are indicated by the size of the dot (scales at the bottom)

Further examination of genes from each cluster in our single-cell sequencing experiment of 3 dpi mock vs LACV-infected COs identified several interferon (IFN)-stimulated genes (ISGs) as differentially expressed between committed neurons (C1) and neural progenitors (C2, C6, C7) (Fig. 7, upper section). Neural progenitor clusters from the LACV CO all contained proportionally greater numbers of cells expressing higher amounts of ISG transcripts relative to mock demonstrating a robust IFN response. By comparison, the cluster containing committed neurons (C1) had a much weaker and less inclusive ISG response suggesting that even the surviving committed neurons had a limited IFN response. To further validate these findings, transcripts of the IFN signaling pathway were also compared between the mock and LACV COs (Fig. 7 lower section). Levels of IFN receptor and downstream signaling effector transcripts were proportionally increased in neural progenitor clusters in the LACV-infected organoid relative to mock. In contrast, the proportions of these transcripts were largely unchanged in the committed neuron cluster when mock was compared to LACV infection suggesting signaling was impaired in the cells. Thus, neural progenitors in the infected organoid mounted a robust IFN signaling and ISG response that was not found in committed neurons.

### Exogenous administration of type I IFN inhibits LACV-induced cell death in COs

**Fig. 8:**
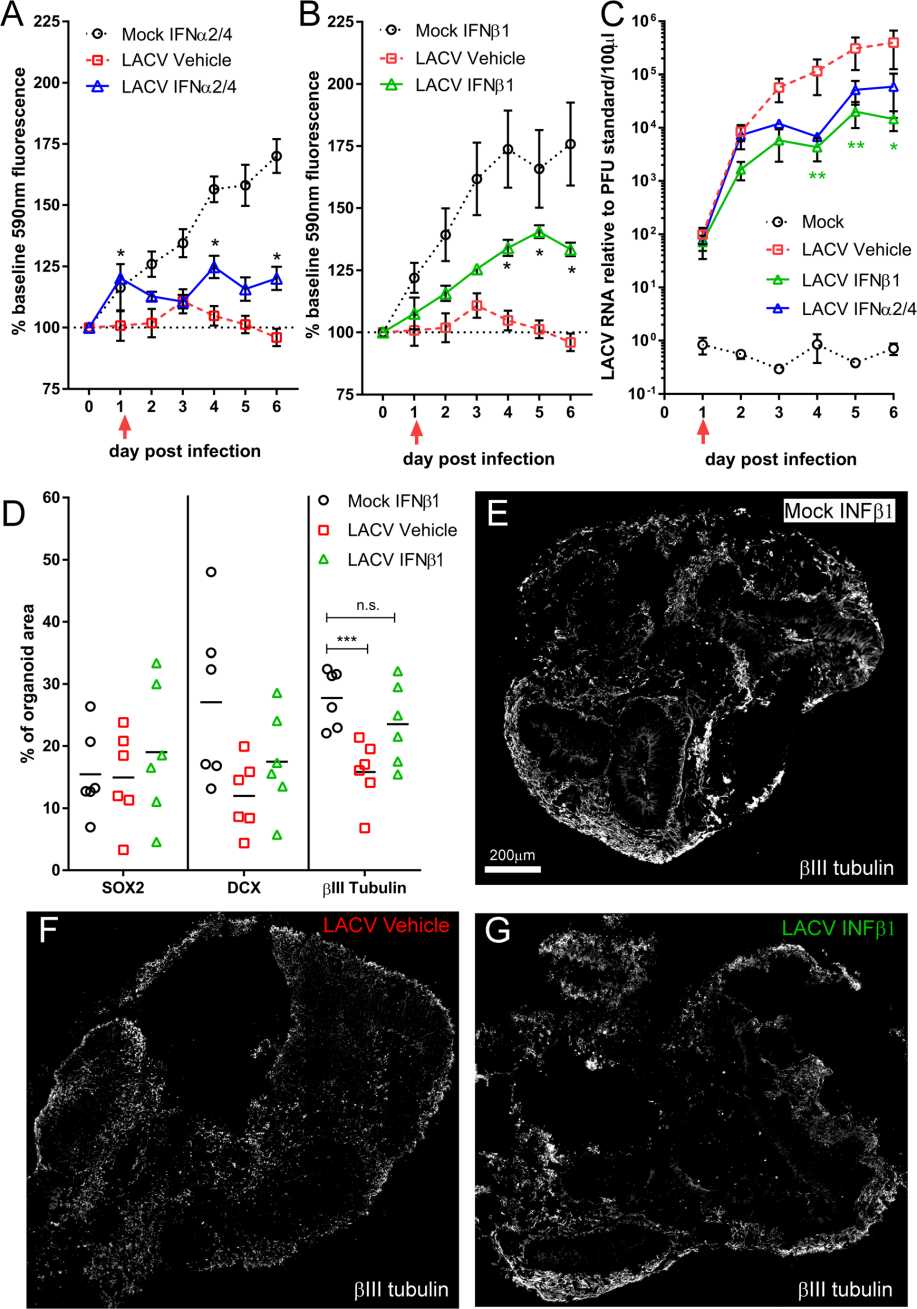
Type I IFN signaling induction in committed neurons increases cell survival. Cell viability of mock- or LACV-infected COs treated with either **a** IFNα2 and IFNα4 concomitantly or **b** IFNβ1 individually were measured using a resazurin reduction-based assay. Data is plotted as an average percent of base fluorescence measured at 590 nm for each CO. Mock IFN-treated samples are indicated by open circles, LACV vehicle-treated samples are indicated by open squares and LACV IFN-treated samples are indicated by open triangles. Fluorescence for each sample at each time point was read in triplicate at the indicated time point. A two-way ANOVA with a Sidak’s multiple comparisons test was used to determine significance. *p < 0.05. Data are representative of *n* = 3 mock IFNα2/4, *n* = 6 LACV Vehicle, *n* = 3 LACV IFNα2/4, *n* = 6 mock IFNβ1 and *n* = 6 LACV IFNβ1 treated COs. **c** Supernatants from mock (black circles), LACV IFNβ1 (green triangles), LACV IFNα2/4 (blue triangles), or LACV vehicle (red squares) treated COs were collected daily and assayed for viral RNA via qRT PCR. Data are presented as LACV RNA expression relative to an experimentally determined PFU standard. A two-way repeated measure ANOVA with a Dunnett’s multiple comparison test was performed on the antilog of the experimental values to establish differences between LACV vehicle and IFN treated samples. **p < 0.01, *p < 0.05. *Color denotes which condition differed relative to LACV vehicle. **d** Quantification of immunohistochemical labeling for Sox2, DCX, and βIII tubulin in sections from the same COs shown in **b** are plotted as a percent positive signal of organoid area. A Kruskal-Wallis one-way ANOVA test with multiple comparisons was used to determine significance. ***p < 0.005. Representative sections of βIII tubulin staining in whole COs treated with **e** mock INFβ1, **f** LACV vehicle, or **g** LACV IFNβ1 are shown

Due to the differential IFN signaling and ISG responses of neural progenitors and committed neurons in LACV-infected COs (Fig. 7b), we exogenously administered recombinant IFN to LACV-infected organoids 24 h post-infection to determine if type I IFN activation affected CO viability. As both α and β IFNs have been shown to induce ISG signaling in neural-lineage cells [35], COs were treated with either IFNα2 and IFNα4 concomitantly (Fig. 8a) or IFNβ1 (Fig. 8b). Both IFNα2/4 and IFNβ1 treatment of LACV-infected COs resulted in a significant increase in viability compared to vehicle-treated infected COs (Fig. 8a, b). However, only IFNβ1 treatment resulted in sustained increased viability throughout the experiment (Fig. 8b). qRT PCR analysis of supernatants from treated COs showed a modest, but significant, 0.5–1 log decrease in LACV RNA at multiple days during the experiment in IFN-treated COs (Fig. 8c). This finding demonstrated that exogenous application of type I IFN induced activation of antiviral signaling during LACV infection.

To determine a cell-type-specific effect of IFN signaling induction, we immunohistochemically labeled sections from mock IFNβ, LACV vehicle, and LACV IFNβ-treated COs with markers of neural progenitors (Sox2), immature (DCX), and mature (βIII tubulin) committed neurons and quantified positive signal as a percent of organoid area (Fig. 8d). On average, neural progenitor Sox2-positive signal was unchanged regardless of infection or recombinant IFN treatment. In contrast, DCX and βIII tubulin expression decreased in the LACV vehicle treatment group relative to the mock IFNβ-treated group, with βIII tubulin being statistically less (Fig. 8d). This further confirmed our previous findings of committed neuron loss with LACV infection in COs (Figs. 5 and 6). However, DCX and βIII tubulin signal was on average higher in LACV IFNβ-treated COs compared to LACV vehicle. Collectively, these data suggest IFN treatment of LACV-infected COs promotes cell survival primarily of committed neurons likely through the activation of the type I IFN signaling and the ISG response.

## Discussion

In monolayer culture, differentiated neurons have been shown to be more resistant to LACV infection-induced apoptosis than undifferentiated neurons [8]. However, this critical question has not been addressed in a complex neural tissue with neurons at various developmental stages. Here, we examined LACV infection in iPSC-derived human COs undergoing neurogenesis. We found LACV infection decreased cell viability and increased apoptosis in COs as early as 3 dpi (Fig. 3). By 6 dpi, structural complexity was reduced (Figs. 1f, and 3d) and cell viability decreased relative to mock (Fig. 3a) suggesting the loss of cells undergoing neural development was sustained during active LACV infection. Apoptosis was observed primarily in developmentally committed neurons and less frequently in neural progenitor cells (Figs. 3, 4, and 5) indicating that maturing neurons were more susceptible to virus-induced death.

COs are a recently developed and powerful model with which to study basic neurodevelopment and human neurological disease. This model utilizes human iPSCs treated with specific growth factors and conditions to stimulate the self-assembly of an organized tissue that contains most cell types found in the developing brain including neural progenitors, committed neurons, micro-, astro-, and oligodendroglia [13, 18, 36–38]. Previous studies have shown that neurons within COs mature and form functional connections which strengthen through time [18, 39]. While it remains undetermined when the earliest of these connections form, significant electrical activity is detected in 2-month-old organoids with spiking activity detected at 6 weeks [40]. Similar with these previous studies, we found significant numbers of committed, mature neurons in COs at 21 days of neural induction (Figs. 4, 5, and 6), suggesting neuronal networks could be forming. This time point is prior to the proliferation of glial cells within the CO which occurs primarily between 30 and 80 days post neural induction [18, 36, 37]. This allowed us to take advantage of this system to specifically examine the response of neuronal cells, from progenitors to mature cells, to LACV infection with minimal confounding influence of glial cells.

Our study contrasts with previous work where differentiated cell were more resistance to LACV-induced apoptosis [8]. However, the cells in this previous study were derived directly from a pluripotent human testicular embryonal carcinoma and induced toward a neural ectodermal lineage [41]. As such, those cells do not undergo a typical developmental neurogenic program and may not express key determinates of susceptibility that would be present in cells of the developing human brain. In contrast, COs undergo a neurogenic program similar to normal human cortical development [13] and contain heterogenous neural-lineage cell types, such as radial glial cells, that provide important trophic and developmental cues [42] that are lacking in homogenous neural cultures [43]. Thus, COs provide a more relevant model of LACV infection in a developing human brain, suggesting that the finding that committed neurons are more susceptible to virus-induced apoptosis may be more accurate than findings from monocultures.

Our single-cell sequencing analysis demonstrated that cells with the transcriptional profile of committed neurons were decreased by LACV infection. However, another undefined population (C3) increased with infection (Fig. 5e). This population did not express transcripts consistent with neural progenitors or committed neurons. However, like the committed neurons cluster (C1), *VIM* expression was minimal (Fig. 5b). *VIM* encodes the intermediate filament vimentin which is typically expressed by neural progenitors, but its expression decreases as these progenitor cells mature and become committed neurons [33]. Thus, this cluster may contain cells that have undergone abnormal transcriptional maturation as a consequence of viral infection, as has been demonstrated with other pathogens such as cytomegalovirus [44]. These cells may be derived from another undefined cluster (C4) that has some *SOX2* and *VIM* expression (Fig. 5b) and also shows a modest proportional decrease in cell number with infection (Fig. 5e). Further analysis of these cells at multiple time points post-infection could provide evidence on how LACV infection alters the transcriptional profile of both neural progenitors and committed neurons.

Our flow cytometry data demonstrate an increase in the proportion of LACV^+^ apoptotic immature and mature neurons in infected COs indicating a cytotoxic effect of virus infection (Fig. 6). However, our histological data (Figs. 3f and 4) and flow analysis (Fig. 6) indicate that not all apoptotic cells are infected suggesting that some cells are dying due to a secondary effect of viral infection. This phenomenon occurs in other in vivo models of viral encephalitis [45–47] and has primarily been attributed to glial and immune activation [48–50]. However, because glial immune responses are largely absent from COs at this developmental timepoint [13, 36], this implies the cytotoxic trigger(s) originate from neural-lineage cells. Developing neurons within COs establish functional neuronal networks [18, 39], the connectivity of which is required to prevent activation of intrinsic apoptotic cellular programs [51]. Thus, uninfected maturing neurons may die as a result of losing connectivity with other infected apoptotic neurons. Alternatively, these neurons may undergo apoptosis following bystander activation of their innate immune responses. Previous studies by our lab and others have shown that neurons undergo apoptosis following toll-like receptor (TLR) signaling through endocytosed viral RNA or micro-RNA produced by dying neurons [52–54]. The environment of infected, dying neurons in the organoids may provide sufficient stimuli to induce TLR-mediated neuronal apoptosis.

While well defined in immune cells, the type I IFN response to virus infection in human neural-lineage cells has not been well characterized. We found that CO neural progenitors, but not committed neurons have a robust IFN signaling and ISG response to LACV (Fig. 7) correlating with resistance to virus-induced apoptosis (Figs. 4, 5, and 6). These findings contrast with two other studies of alphavirus infection of differentiated neurons in monolayers, where enhanced type I IFN responses in differentiated neural-lineage cells correlated with increased resistance to virus-induced cytopathy primarily in differentiated neurons [35, 55]. Our data clearly indicate that transcripts for IFN signaling and ISGs are primarily induced in neural progenitor cells compared to committed neurons in response to LACV infection (Fig. 7). Although the difference in these studies could be due to the systems used, i.e., monolayers versus organoids, it is also possible this differing response may be due to specific actions of the virus. For example, LACV has been shown to inhibit IFN production by neurons in an in vivo mouse model through actions of its non-structural protein NSs [56]. Possibly, LACV may suppress innate immune IFN signaling in committed neurons more than in neural progenitor cells leading to enhanced apoptosis in this population.

Our data also demonstrate that exogenous administration of recombinant IFN to LACV-infected COs rescues viability (Fig. 8a, b). Because most of the cell death associated with LACV infection was in committed neurons, we reasoned the increase in viability is most likely due to enhanced survival of this cell type. We confirmed this by immunohistochemical quantification of whole sections of treated and untreated LACV- and mock-infected COs (Fig. 8c). Previous studies using IFNs as a treatment in the CNS have shown some negative consequences including inhibition of neurogenesis and promotion of apoptosis when administered at high concentrations [57]. However, IFNβ1 can also protect cells in the brain from apoptosis in the absence of growth factors [58]. Our current data indicates that induction of IFN signaling and the ISG in committed neurons correlates with increases cell survival during LACV infection and should be further examined as a potential therapeutic for the treatment of viral encephalitis.

## Conclusions

Determining the effect of virus infection in the brain on neuronal subpopulations is critical to our understanding of encephalitic disease. Here, we demonstrate that cerebral organoids are a useful model to study these effects as they contain neurons at multiple developmental stages patterned similarly to a human brain. Experimentally, we show that committed neurons are more susceptible to LACV-induced apoptosis than neural progenitors despite similar levels of infection. This susceptibilty can be explained, at least in part, by the poor type I IFN response we observed in committed neurons compared to progenitors during virus infection. Furthermore, we demonstrate that CO cell viability can be rescued by induction of type I IFN signaling using recombinant IFNs. Together these findings demonstrate that induction of IFN signaling is a critical determinant in encephalitic virus-induced neuronal death.

## Data Availability

The sequencing dataset generated and analyzed during the current study will be made publicly available through NCBI GEO (accession#GSE131434) upon publication. Currently, the dataset can be released through NCBI GEO on a reasonable request to the corresponding author.

## Abbreviations

CCA: Canonical correlation analysis
CO: Cerebral organoid
DCX: Doublecortin
EB: Embryoid body
IFN: Interferon
iPSC: Induced pluripotent stem cell
ISG: Interferon stimulated gene
LACV: La Crosse virus
PFU: Plaque forming unit
qRT: Quantitative reverse transcription
TLR: Toll-like receptor
tSNE: t-distribution stochastic neighbor embedding
βIII tubulin: Class III β-tubulin
